# Why advanced therapy medicinal products struggle in clinical translation: an in-depth analysis of developmental challenges in the EU

**DOI:** 10.3389/fmed.2026.1839531

**Published:** 2026-06-02

**Authors:** Charlotte Van Isterdael, Edurne Mugarza, Piret Fischer, Koen Debackere, Peter Vandenberghe, Silvia Martin Lluesma, Johan Van Eldere, Isabelle Huys

**Affiliations:** 1 Department of Pharmaceutical and Pharmacological Sciences, KU Leuven, Leuven, Belgium; 2 Scientific Management Area, Vall d’Hebron Institute of Oncology (VHIO), Barcelona, Spain; 3 Department of Emerging Technologies, Fraunhofer ISI, Karlsruhe, Germany; 4 Laboratory of Experimental Hematology, Department of Oncology, KU Leuven, Leuven, Belgium; 5 Laboratory for Genetics of Malignant Disorders, Department of Human Genetics, KU Leuven, Leuven, Belgium; 6 Department of Basic Medical Sciences, Faculty of Medicine, San Pablo-CEU University, CEU Universities, Madrid, Spain; 7 European University Hospital Alliance (EUHA), Brussels, Belgium

**Keywords:** advanced therapy medicinal product (ATMP), cell and gene therapy (CGT), clinical translation, regulatory challenges, scoping review

## Abstract

Advanced Therapy Medicinal Products (ATMPs) can offer unprecedented therapeutic benefit for patients with limited treatment options. However, their clinical translation and early-stage clinical development remain highly complex, particularly for academic developers and small- and medium-sized enterprises (SMEs). This study presents a comprehensive analysis of the different types of challenges that ATMP developers face in initiating clinical trials, based on a scoping literature review, conducted according to the JBI methodology for scoping reviews and reported according to PRISMA-ScR. PubMed, Embase and Scopus were searched for peer-reviewed literature covering the last decade, focusing on publications discussing regulatory challenges for ATMP developers at the stage of clinical trial initiation. Our findings show that key challenges exist in four areas, comprising (a) preclinical evidence and product characteristics, (b) cell sourcing and manufacturing, (c) clinical trials and (d) regulatory landscape. Early engagement with regulators and the availability of up-to-date guidance documents emerged as key strategies to align developer efforts with regulatory expectations, while the review also identifies practical recommendations to support more efficient and harmonized clinical translation. In line with the objectives of JOIN4ATMP, these insights are valuable for developers and informative for regulators to foster more efficient and aligned ATMP development pathways.

## 1 Introduction

Advanced Therapy Medicinal Products (ATMPs) are medicines based on genes (gene therapy medicinal products (GTMPs)), cells (cell therapy medicinal products (CTMPs)) and tissue-engineered products (TEPs) (1). ATMPs represent promising treatments for conditions with limited or no existing therapeutic options, especially in rare genetic, neurodegenerative and autoimmune disorders, hematological and solid malignancies, and orthopedics (2). Patients in the European Union (EU) should have safe, efficient and equitable access to ATMPs. In 2008, in light of the striking advancements in the field, the specific regulation on ATMPs (EC No 1394/2007) was introduced at the EU level (1).

Despite this dedicated framework, access to ATMPs across the EU remains limited. Nineteen years after the introduction of the ATMP regulation, only 29 products have received marketing authorisation in the EU, including 21 GTMPs, 4 CTMPs and 4 TEPs, with only a minority successfully reaching patients (2, 3). Although a vast amount of basic research is ongoing, a substantial gap remains between preclinical development and clinical application, a challenge commonly referred to as “the translational gap” in ATMP development (4–7). The clinical translation of ATMPs is significantly more challenging than the development of conventional pharmaceuticals due to their particular complexity, high development costs, and the need for highly specialized expertise (7). Moreover, many ATMPs target small, fragmented populations or require personalized approaches, offering limited commercial incentives and thus often falling outside the interest of large pharmaceutical companies (2, 7–9). As a result, ATMP development is often situated in academic institutes or small- and medium-sized enterprises (SMEs). Although several publications have discussed challenges in ATMP development, these studies typically focus on specific phases of the development chain or are based on narrative reviews. A comprehensive review using a structured methodology to map regulatory challenges across the full ATMP development chain is currently lacking. In this context, this review classifies challenges across different domains, including preclinical evidence and product characterization, manufacturing and cell sourcing, clinical trial design and conduct, and regulatory processes, with regulatory considerations shaping and intersecting with each of these areas. This scoping literature review includes publications from multiple jurisdictions (EU, US, Japan and Canada), but the analysis and interpretation are primarily focused on the European regulatory context, where specific structural and regulatory features may influence how these challenges are experienced and addressed. This geographical focus is particularly relevant in light of the well-described “Atlantic divide” in ATMP development, with a higher number of authorized cell and gene therapies in the US (49 products) (10) than in the EU (31 products) (11). Importantly, the US number only accounts for cell and gene therapies, so the total approved ATMP number might be even higher. By identifying frequently reported challenges developers face, the study seeks to improve regulatory awareness and understanding among academic and SME developers by highlighting frequently emerging challenges during development that may impede successful translation at later stages.

## 2 Methods

### 2.1 Guidelines

To assess the challenges perceived by ATMP developers for the initiation of clinical trials, a scoping literature review was conducted. The review followed the JBI methodology for scoping reviews and the PRISMA extension for scoping reviews was used for reporting (Supplementary materials I) (12). A protocol was submitted to the Center for Open Science before the selection of sources of evidence started (13).

### 2.2 Data sources and search strategy

A comprehensive search was conducted using three scientific databases, being PubMed, Embase and Scopus. The search string (Supplementary materials II) was designed with the assistance of a medical librarian of KU Leuven, to ensure it is comprehensive and sensitive to the relevant literature. Search terms consist of a combination of three different concepts (ATMPs, developers and regulatory) with synonyms and index terms for PubMed and Embase (Mesh-terms and Emtree terms, respectively). A search validation procedure was employed, validating if some key relevant articles were identified through the search strategy.

### 2.3 Eligibility and article selection

After de-duplication of the retrieved articles from PubMed, Embase and Scopus, articles were selected for inclusion. Selection of sources of evidence was performed using pre-defined eligibility criteria, as presented in Table 1. To ensure consistency and to test the eligibility criteria, an unblinded pilot title and abstract (tiab) screening was conducted by two independent researchers, using the same 50 articles. These reviewers then conducted a blinded pilot tiab screening of 200 articles. Subsequently, the remaining articles were screened by the two researchers. De-duplication and tiab screening were conducted using the software Rayyan. For full text screening, a pilot screening of the same 5 articles was followed by full text screening of all articles included after tiab screening.

**Table 1:** Eligibility criteria for selection of articles.

Eligibility criterium	Inclusion	Exclusion
Paper type	Peer-reviewed articles	Conference abstracts, books, not peer-reviewed articles
Publication year	Articles published in 2014 or later	Articles published before 2014
Therapy type	Articles related to ATMPs.	Articles related to other (innovative) therapies.
Regulatory aspect	Articles regarding the regulatory process of clinical trial initiation	Articles without a regulatory aspect
Geographical region	Articles regarding an EEA-country, US, Japan or Canada	Articles regarding another country
Language	Articles written in English	Articles written in another language

### 2.4 Data extraction, analysis and synthesis

Data from all included articles were extracted using a standardized data extraction form, including both predefined criteria, based on relevant literature and researchers’ discussions regarding the research question, as well as new themes that emerged during the extraction process. To pilot the form, two reviewers independently extracted data from three articles.

Data extraction was carried out by two reviewers: each reviewer extracted data from half of the included articles and cross-checked the data extracted by the other reviewer to ensure accuracy and completeness. Both descriptive parameters (e.g., article title, author name, publication year) as well as content-related parameters (challenges perceived by ATMP developers) were extracted from the included articles. Data extraction was performed in Microsoft Excel, followed by thematic analysis and narrative synthesis to identify key regulatory challenges. The results were divided into an overview of included studies and challenges in clinical translation of ATMPs.

## 3 Results

### 3.1 Overview of included articles

**Figure 1:**
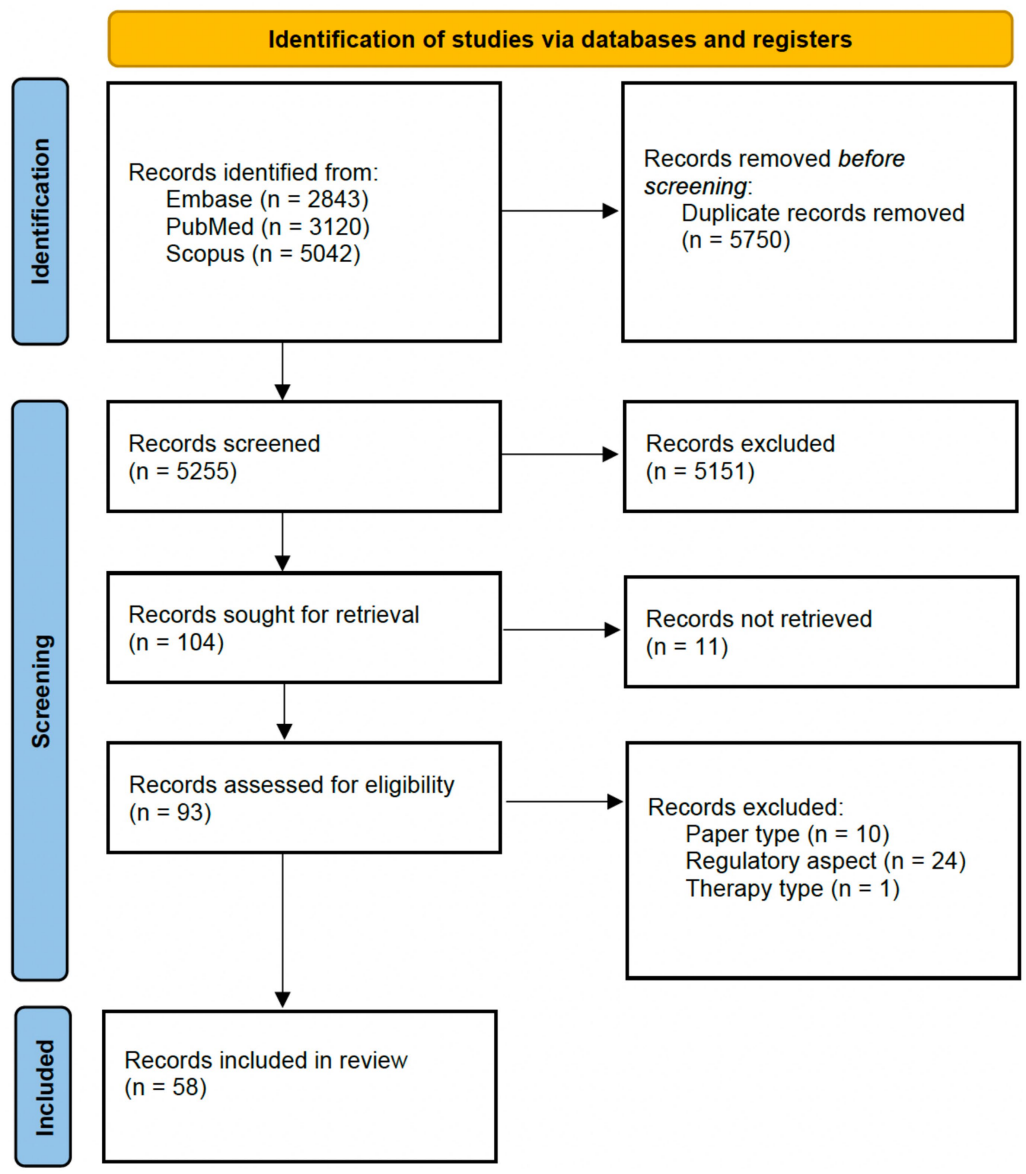
PRISMA flowchart of selection of sources of evidence.

**Figure 2:**
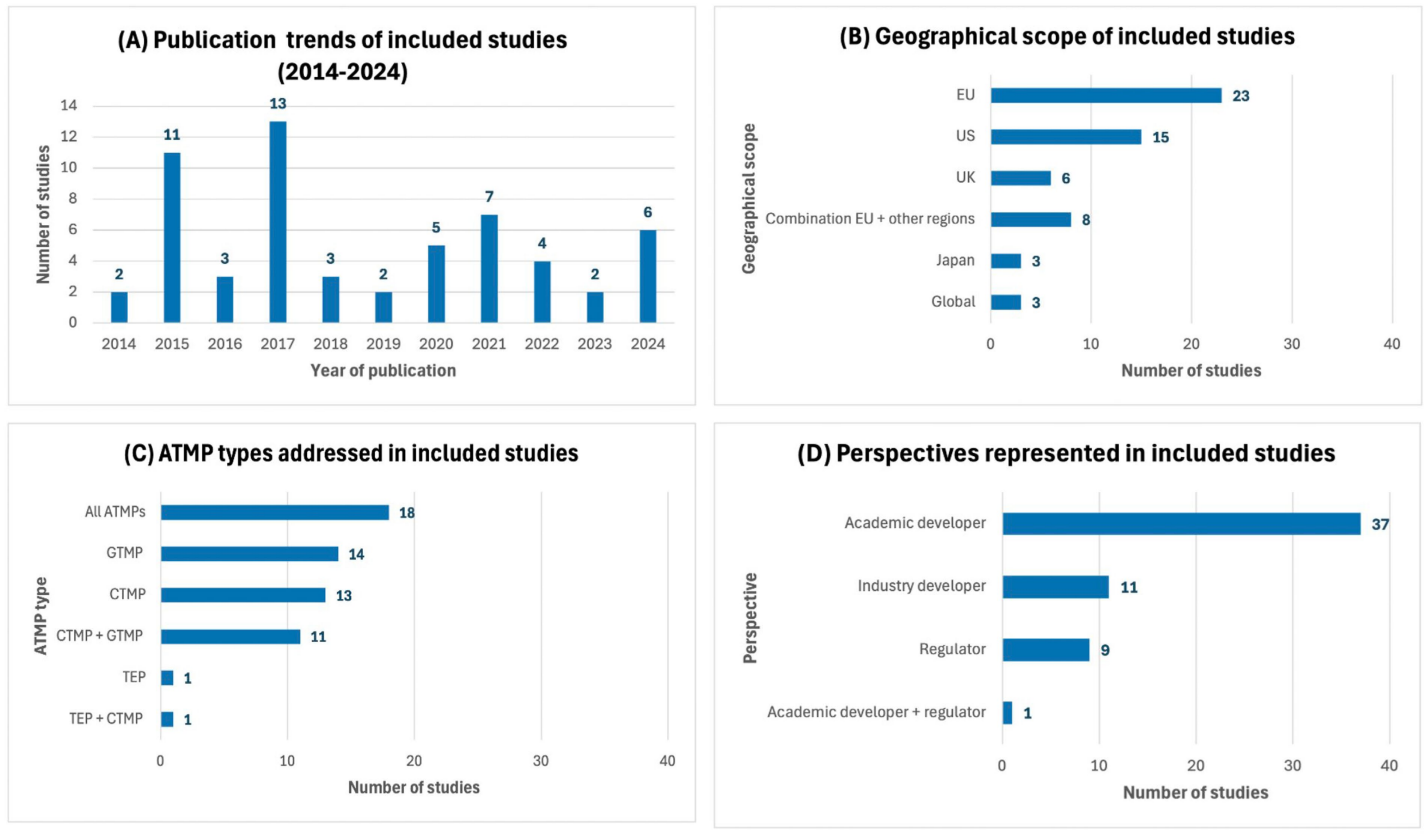
Overview of included studies. Panel **(A)** shows publication trends between 2014 and 2024. Panel **(B)** presents the geographical scope of the studies, with most focusing on the EU or the US. Panel **(C)** summarizes the ATMP types addressed in the articles, with most studies covering all ATMP types, and approximately equal numbers of articles covering GTMPs or CTMPs. Panel **(D)** depicts perspectives represented. The literature was largely driven by an academic developer perspective, with fewer contributions from industry or regulatory stakeholders. EU, European Union; US, United States; UK, United Kingdom; ATMP, Advanced Therapy Medicinal Product; GTMP, Gene Therapy Medicinal Product; CTMP, Cell Therapy Medicinal Product; TEP, Tissue Engineered Product.

As presented in the PRISMA flowchart (Figure 1), a total of 58 articles were considered for data extraction. Detailed characteristics of the included articles, comprising the title, authors, publication year, study design, and geographical scope, are presented in Appendix 3. Distribution of the included studies according to year of publication, geographical scope, ATMP type and perspective is presented in Figure 2. The majority of included studies were reviews (*n* = 44), followed by empirical research studies (*n* = 7; including surveys and focus group discussions), perspectives (*n* = 6), and one document analysis.

### 3.2 Challenges in clinical translation of ATMPs

**Figure 3:**
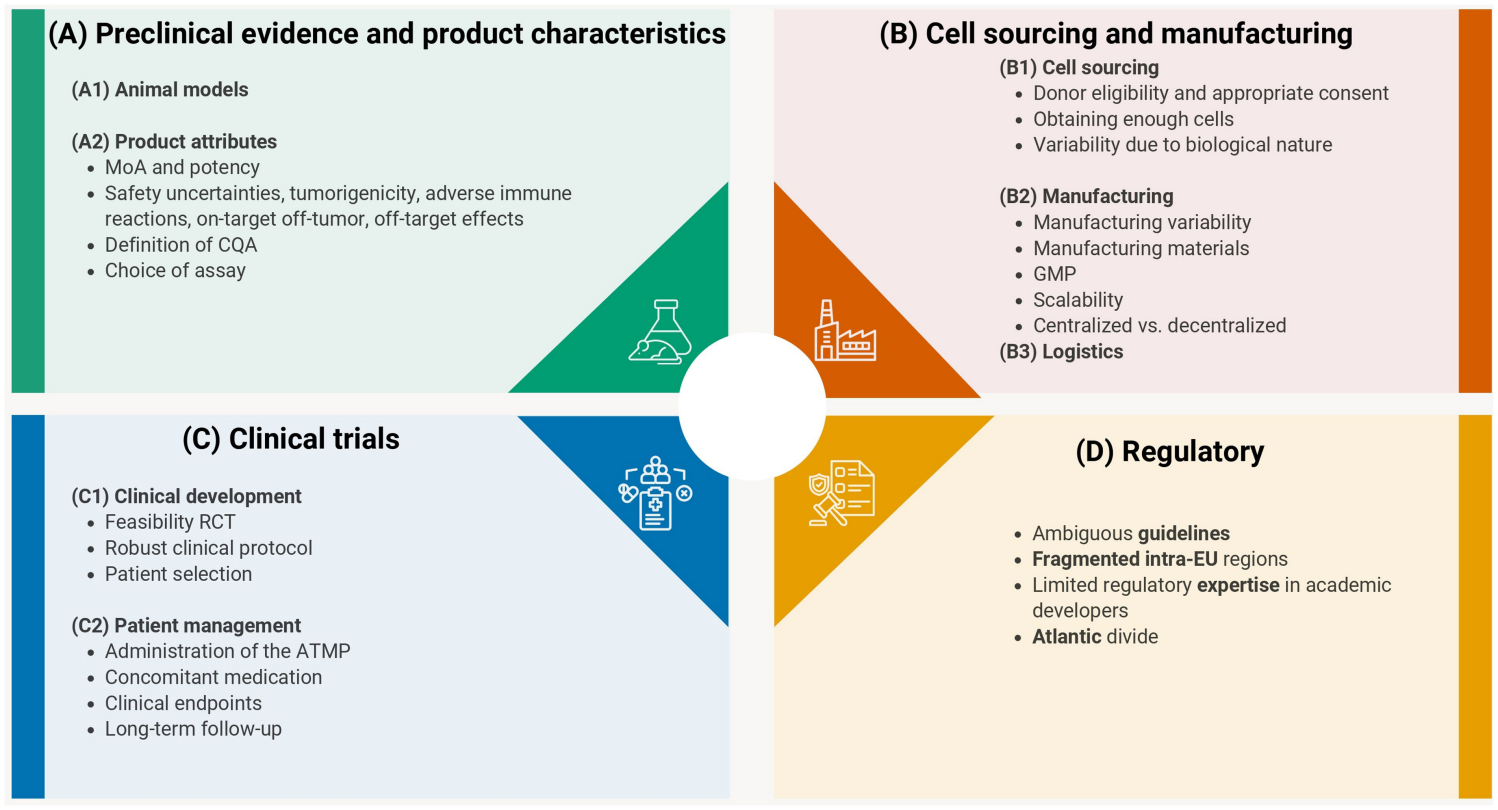
Overview of key challenges encountered during the clinical translation of ATMPs, spanning **(A)** preclinical evidence and product characteristics, **(B)** cell sourcing and manufacturing, **(C)** clinical trial feasibility and management, and **(D)** regulatory considerations. ATMP, Advanced Therapy Medicinal Product; CQA, critical quality attributes; MoA, mechanism of action; RCT, randomized clinical trial.

The clinical translation of ATMPs faces unique challenges, related to the complex and individualized nature of these therapies. Figure 3 provides a visual representation of the different categories of translational challenges encountered on the path from preclinical development to clinical trial initiation. Importantly, many of these challenges require consideration during the pre-clinical and translational phase to ensure that, at the time of clinical trial application (CTA), all regulatory requirements are met, and delays or negative outcomes in the authorization process are avoided. The following sections further elaborate on each of these challenges and propose potential solutions if identified in the scoping review.

(A) Preclinical evidence and product characterization

1 Animal models

In the CTA, ATMP developers are required to provide pre-clinical data, generally generated in animal models. However, animal models only poorly mimic human physiology and immune responses, and the human-derived nature of ATMPs often necessitates immunocompromised animals, further reducing their relevance (14–24). While homologous animal models can offer proof-of-concept insights, cellular and immunological differences restrict their predictability for key parameters such as viability, functionality, persistence, dosing and toxicity (14–17, 20, 24–30). This, and the increasing need to apply the 3Rs principle, which encompasses Replacing, Reducing and Refining the use of animal models in research, requires a paradigm shift in the approach to pre-clinical testing needed for the CTA of ATMPs. According to literature, this shift comprises a complementary strategy combining *in vivo* approaches with alternative *in vitro* methods such as human cells or human cell lines, in silico analyses, human-on-a-chip platforms, cell cultures and organoids (24, 28, 29, 31–33).

2 Product attributes

As with conventional drugs, regulatory bodies require an extensive characterization of the product under development, and characterizing a clear mechanism of action (MoA) is of utmost importance to ensure product safety (18, 22, 34). However, three studies reported that the MoA of many ATMPs is complex, pleiotropic, not necessarily linked to the nature of the product, and therefore often poorly understood (18, 34, 35). Furthermore, obtaining reproducible potency was described to be challenging due to the variability in manufacturing materials and processes, as well as the complexity of scaled manufacturing [further discussed in (B-2) manufacturing] (35–37).

As reported in one article, uncertainty regarding safety of ATMPs is influenced by the biologic variability of these products, including variability and variation in the starting material, intrinsic product complexity and the dynamic heterogeneity of living cell populations (26). Moreover, tumorigenicity is a major concern, as the administration of an ATMP can contribute to tumorigenicity through several different mechanisms. Insertional mutagenesis (IM) after GTMP administration was described the most (*n* = 10 articles), especially prominent in pediatric patients with decades of life, and thus exposure, ahead. Integrating vectors can insert into the host genome, potentially activating neighboring oncogenes or inactivating tumor suppressor genes, which may lead to cancer development years after treatment (17, 21, 23, 25, 31, 35, 38–40). The risk of IM depends on several factors, including the vector design and integration profile, vector or cell dose, the transgene product, and the target cell population or organ, but it can be reduced through improved vector design as well as incorporating safety modifications (38, 39). Although non-clinical integration studies for candidate GTMPs are expected by regulators, one article reported that it remains unclear for some developers how regulatory bodies will address this risk, given that integration events occur largely at random (41). In addition to IM, off-target double-stranded breaks induced by gene editing tools can also contribute to tumorigenicity, even when no viral vectors are used (23). The third described tumorigenicity mechanism may occur with use of progenitor or non-terminally differentiated cells, which is associated with teratoma development in recipients (14, 42–44).

Furthermore, adverse immune reactions are common in ATMPs, and arise from distinct but interrelated mechanisms (14). Firstly, immune-privileged sites like the eye are susceptible to inflammation disrupting tissue barriers, as described for retinal administration of gene therapy, which can elicit intra-ocular inflammation and antidrug antibodies (45). Secondly, the Jesse Gelsinger case illustrates the potential for massive systemic responses to non-self components in the product, such as the viral vectors (35). Similarly, two included articles described specific immunogenic challenges for xenogeneic components in murine-derived chimeric antigen receptors (CARs), which can provoke human anti-mouse immune responses, requiring mitigation approaches such as humanized or fully human antibodies (25, 31). Donor-recipient immune matching of transplanted cells and biomaterials can reduce inflammatory recognition and elimination, but it may not fully prevent it (14). As a result, immune suppression is often required together with ATMP administration to modulate host immunity.

Lastly, as described in several articles (*n* = 5), on-target off-tumor as well as off-target effects, particularly in CAR/TCR-therapies, represent a central safety limitation, which can be mitigated through targeted delivery of the genes to the intended tissue or by performing extensive specificity and off-target screening using predictive tools in advance (21, 25, 31, 33, 46, 47).

Multiple included articles (*n* = 7) reported that ATMP developers often struggle to define product Critical Quality Attributes (CQAs) and critical process parameters due to the lack of quality standards and the resulting strict product qualification expectations from national regulators (17, 18, 44, 48–51). Consequently, international standardization of quality control is essential, as it is being done in the US, where the FDA, together with Standard Development Organizations (SDOs) are working on consensus standards (16). According to Silverman et al., ATMPs typically require dozens of release assays to address complex MoA and safety risks, making assay establishment a substantial undertaking (36). Moreover, ATMPs often require development of new, fit-for-purpose assays, with rigorous risk assessment and early communication with regulators (18, 31, 46, 52, 53). Ideally, these assays are developed early to confirm product activity, guide dose selection, and enable comparability at commercial scale. Yet, the product complexity often entails evolving insights, hence assays and standards co-evolving with the product (18).

(B) Cell sourcing and product Manufacturing

1 Cell sourcing

Results reported in five included studies indicate that suitable donors for allogeneic therapies can be difficult to identify due to strict eligibility criteria and indication-specific testing requirements under Directive 2004/23/EC on human tissues and cells (14, 18, 36, 54, 55). Moreover, although many cells and tissues are stored in biobanks, the original consent does often not cover clinical or commercial use (14).

Additionally, obtaining sufficient amounts of starting material for manufacturing can be challenging, due to disease-related deficits (e.g., HSCs in HIV-infected individuals) or tissue-specific shortages (e.g., fetal tissue for dopaminergic cell replacement in Parkinson’s disease) (29, 34, 38). Suggested approaches in literature comprise improved protocols for collection and isolation of cells and directed differentiation of stem cells, e.g., differentiation of transplantable dopaminergic progenitor cells from human Pluripotent Stem Cells (hPSCs) (29, 34).

As described in several articles (*n* = 11), the biological nature of ATMPs introduces inherent variability in the starting material, therefore generating variability in manufacturing as well as among products (24, 31, 35, 46, 47, 51, 55–59). In autologous therapies, patient-specific factors such as prior treatments and disease status drive variability (31, 55). In allogeneic starting material, donor variability is the primary cause of batch-to-batch variability (31, 37, 55). Moreover, heterogeneity in clinical apheresis unit practices further compounds this variability, constraining manufacturers’ ability to control starting-material quality via standardized processes (46, 55). While variable quality and quantity in starting material cannot be eliminated, the risks associated should minimized by understanding its origin, and by confirming required properties with validated analyses (36).

2 Manufacturing

i. Manufacturing variability (process/technical variability)

Some studies (*n* = 7) highlighted variability of manufacturing processes as a challenge when moving into clinical development, leading to product variability and thus variation within and among trials (31, 36, 47). The manufacturing process of ATMPs is a multi-step and complex process, and often relies on manually operated systems and protocols lacking clear step-by-step-definitions, which makes the product vulnerable to inter-operator variability (20, 24, 36, 51). The variability in starting biological material, previously described in B-1, further contributes to manufacturing variability (24, 46). Five studies reported mitigation strategies such as closed, automated systems (see “Scalability”), as these can improve process robustness, or detailed protocols combined with digitally tracking all recorded information (24, 36, 47, 51).

ii. Manufacturing materials

Another major challenge in manufacturing ATMPs, as reported in 12 articles, regards the material used during the manufacturing process. First of all, many current processes use undefined or xeno-derived products, which could, even with defined purity specifications and thresholds for “acceptable contamination,” result in batch-to-batch variability as well as in zoonotic transmission of adventitious agents (e.g., Bovine Spongiform Encephalopathy BSE) or immune complications once infused into the patient (18, 21, 22, 26, 36, 39, 44, 57, 60). Consequently, serum-free or serum-replacement media are strongly recommended for clinical applications (22, 60). However, as suitable alternatives are still lacking for certain products, particular attention must be paid to appropriate sourcing, traceability and virus clearance to comply with regulatory standards (18, 36, 54, 57, 60). Secondly, certain raw materials used in pre-clinical research needed for the manufacturing of ATMPs are not certified for human use, or clinical-grade alternatives are unavailable or more expensive (22, 38, 48, 57).

iii. Good manufacturing practices

The most frequently reported challenge in this review is GMP-compliance when moving into clinical development, as regulators generally expect critical preclinical studies to be performed using the final product manufactured under an appropriately GMP-qualified process (14). GMP-related challenges identified in this scoping-review include complexity, expertise, access to facilities and related costs.

For small academic developers, it remains complex to comply with GMP in pre-clinical development, and unclear to what extent, and from which stage of the production process, GMP should apply (53). For example, several articles noted uncertainty about whether GMP controls are needed during the early generation of iPSCs (14, 54). Consequently, protocols often need to be modified at clinical translation (29, 51). Additionally, one study mentioned that given evolving genetic or clinical insights, locking in on one GMP-qualified source or platform too early can be risky, as switching later entails repeat testing, with added costs and delays (14). International differences further influence the GMP landscape. GMP facilities in the US that manufacture products for phase I/II and phase II trials are not subject to routine regulatory inspection, and burden for GMP compliance is lower, resulting in an Atlantic divide, introduced above and further discussed in (D) (48). The ATMP-specific EU GMP guideline adopts a risk-based approach, allowing some flexibility in how GMP principles are implemented, provided that product quality, safety and traceability are ensured (18). In addition, GMP-specific expertise in many European, academic groups remain limited (18, 51, 61).

The most frequently reported GMP-related challenge for academic or SME developers, discussed in 18 articles, concerns access to GMP-compliant manufacturing facilities, particularly the major upfront costs to meet stringent quality, regulatory and technological requirements for these sites (14, 15, 18, 24, 47, 48, 51, 59, 61–63). In addition, manufacturing itself is a major cost driver, encompassing GMP-grade raw materials, consumables, equipment, cleanroom operation, personnel, extensive product testing and long culture times (14, 19, 22, 24, 29, 34, 47, 51, 61, 62, 64). This hinders new research centers from entering the field, which makes ATMP development increasingly reliant on specialized GMP centers (15, 48, 51, 62).

Three articles highlighted that several of these manufacturing costs can be reduced and better controlled by expanding in-house testing capabilities to certify raw materials, meet quality standards, and decrease dependency on external suppliers, although this approach is often only feasible for large developers. Costs can also be mitigated through automation, as discussed below, and the use of pre-GMP facilities to develop and optimize robust manufacturing processes (19, 24, 44).

iv. Scalability

Most processes developed in pre-clinical academic settings remain manual and single batch, making production labor-intensive and costly (20, 24, 25, 56). Scaling up for clinical studies requires the manufacturing of these therapies on a larger scale. Closed and automated processes have been proposed in the literature to improve process robustness and support GMP compliance during scale-up. However, their inflexibility and high acquisition and operating costs may limit their implementation, particularly at early development stages and in academic settings (24, 51). Biotechnology and pharmaceutical companies entering the ATMP field should accelerate automation and drive down costs (25, 37). Alternatively, one study proposes conducting clinical development for phase I material using a manual, semi-closed process that can be transferred to a scalable, closed, and automated manufacturing platform if the FIH trial proves successful (56).

Collaborative models between developers and regulators, together with open-access procedures, are considered key to reducing the administrative burden on small academic ATMP manufacturers and facilitating efficient scale-up (14, 29, 35, 49).

v. Centralized vs. decentralized manufacturing

Even at early stages of clinical development, decisions regarding manufacturing strategy can influence trial feasibility, particularly when multiple sites or geographically dispersed patient populations are involved. One article reported that, looking ahead, decentralizing manufacturing is expected to broaden global access by bringing production closer to patients that currently have no access (47). However, reproducing the complex multiple-step processes across sites is challenging, raising costs and prolonging staff training (30). On the other hand, centralized models may offer homogeneity but pose logistical burdens, with increased costs to ensure the cold chain, and more potential quality impacts (51). Overall, both centralized and decentralized approaches present advantages and limitations, and the choice between them will depend on the specific product characteristics, regulatory context, and patient population involved.

3 Logistics

Throughout the whole supply chain, conditions must be tightly controlled, as these biological products are extremely vulnerable to fluctuations in temperature, humidity, shock, and light (32, 36, 47). For this reason, three articles propose the use of intelligent transport systems that continuously monitor these parameters to safeguard product quality (32, 42). In addition, many ATMPs, especially when stored fresh, have a short shelf-life, meaning that, to ensure the quality and efficacy of the product, the manufacturing process must flow seamlessly into administration to the patient (32, 37, 38, 61). A frequently discussed strategy to address this is cryopreservation, which allows longer storage and easier transport, which is particularly helpful in centralized manufacturing models, for treatments requiring multiple administrations, or when administration occurs during surgery (32, 44, 51, 56, 61). However, it requires strictly validated protocols to ensure that product integrity is maintained (61). Additionally, formulation and delivery methods also pose logistical challenges and administration techniques are often novel, complex and not yet standardized (14, 32, 36). Finally, transportation of human material is strictly regulated, and cross-border transport may become logistically complex (14, 59).

(C) Clinical trials

1 Clinical development

Several challenges related to clinical development were identified. First of all, while randomized controlled trials (RCTs) remain the standard level of evidence required for regulatory assessment, this is not always feasible for ATMPs (62). Reasons were described in several included articles (*n* = 7), and comprise very small patient populations, invasive procedures, the absence of alternative treatment options, and the involvement of patients with life-threatening conditions (19, 42, 45, 62, 65, 66). Three other articles described alternatives, including adaptive trial designs, including small, single-arm, short-term, early-phase clinical trials, as well as single-case designs where the patient serves as their own control, and non-inferiority approaches (42, 45, 62).

Secondly, developing a robust clinical protocol for ATMPs can be challenging (36, 50, 64, 67). Therefore, close collaboration with key opinion leaders and regulators is essential to address these complexities effectively (36).

The third challenge concerns patient selection. This process is particularly challenging for FIH trials involving ATMPs, as there is often no established standard for the indications and populations targeted by these therapies (36, 50, 68). Important considerations in patient selection were described in included articles. For example, differential susceptibility to vectors may exist among specific populations, such as pregnant women, children and the elderly. In addition, first-in-human trials raise complex questions such as whether to include severely ill patients, patients with acute or chronically conditions, and whether to involve groups that have historically been underrepresented from research, including pregnant women, the elderly, minority groups, and those with rare conditions (68). In addition, the potential risk of autoimmunity and inflammation raises questions about whether to exclude patients with active autoimmune disorders (25). These decisions require careful discussion and agreement with key opinion leaders (36).

2 Patient management

Across three included articles, dose optimization in FIH trials is described as a major challenge, since ATMPs do not follow traditional animal-to-human dose paradigms and starting doses often rely on prior clinical experience with similar products (23, 35, 41). Their administration adds further complexity as grafted cells can expand *in vivo* and migrate unpredictably to target sites depending on the delivery route, making effective dosing difficult to anticipate (27, 34, 47). Moreover, ATMP administration is frequently invasive and requires specialized equipment, rendering it substantially more complex than for conventional medicines (42, 65).

Another challenge is concomitant medication or, if necessary, immunosuppression, which introduces the risk of interactions between the ATMP and the other treatments (41, 52). Moreover, it often remains unclear how long such immunosuppressive treatment should be continued, creating further uncertainty in the clinical management of patients receiving ATMPs (52).

Determining appropriate clinical endpoints is challenging, as it can be difficult to distinguish the clinical effect attributable to the investigational product from effects related to variations in clinical care or surgical techniques, from the heterogeneity due to patient morbidities, or in situations where the therapeutic effect is not observable for several years (16, 42, 45, 67). Furthermore, for new indications, standard endpoints are often not available, complicating endpoint selection (19). Individual hard endpoints also tend to lack statistical power when patient numbers are limited, which is a common issue in ATMP development (52). As a result, alternative approaches are frequently considered. Composite endpoints, combining biological and functional measures, may offer a more feasible solution, while surrogate endpoints can also be used (52, 62). However, results based on surrogate endpoints may be unreliable if the surrogate has not been properly validated, making it essential to ensure sufficient data and documentation to support the alternative method and to minimize the risk of rejection by regulatory agencies (46, 62).

Lastly, long-term follow-up is essential in ATMP clinical trials, particularly because animal models are poorly suited for robust safety assessment due to the long observation periods required, limited translatability, and difficulties in characterizing potential risks (see A-1) (17, 24, 43, 50, 65). Adverse effects of conventional medicines often diminish once treatment is discontinued because the drug is metabolized and cleared from the body. In contrast, ATMPs involve living cells or biological material that may persist, divide, continue to function, or even integrate into the genome (see A-2) (30, 62). In addition, tissue samples are often required before, during, and after treatment to adequately assess safety and efficacy (64). In practice, obtaining such longitudinal samples can be technically or ethically challenging, and uncertainties around the feasibility of these regulatory requirements may delay clinical trial initiation or, in some cases, prevent these studies from proceeding. ATMP trials also require special monitoring considerations, including immunogenicity, the duration of product persistence in the body, shedding, the possibility of clonal outgrowth, and potential effects on linear growth and maturation in pediatric patients (35).

(D) Regulatory

The conventional regulatory model used for standard pharmaceuticals is not ideal for ATMPs, which would benefit from a more flexible risk-based assessment due to their complex and highly variable nature (14, 15, 27, 29, 41). The EMA has developed and periodically updates guidelines to support ATMP developers navigating this regulatory landscape (17, 26, 27, 29, 41, 46, 53, 59). However, in practice, current standards are often ambiguous or difficult to interpret. Differing perspectives among regulatory authorities contribute to uncertainty, creating a complex regulatory landscape that imposes substantial logistical and financial burdens on ATMP developers (15, 26, 53, 59).

Geographical differences further complicate the regulatory landscape. Within the EU, the translational pathway is governed by numerous legal instruments operating at different levels, and many regulatory challenges arise at the level of individual Member States (15, 19, 27, 42, 43, 46, 49, 58, 61, 67). National differences in GMP requirements, clinical trial approvals, interpretation of hospital exemption, expertise with ATMPs, and development infrastructure, highlights the need for harmonization (15, 19, 27, 42, 43, 49, 58, 61). For genetically modified ATMPs, developers in many EU member states must also obtain a GMO license before initiating clinical trials, but the underlying EU directives have been reported as outdated, inconsistently applied across member states, and associated with long, heterogeneous review processes that are often considered an unnecessary burden (17, 19, 29, 31, 35, 59, 61).

The consequences of these regulatory inconsistencies include inadequate recruitment rates, which is particularly problematic for orphan diseases, difficulties in enabling materials and biologics to cross international borders, and patients traveling to jurisdictions where ATMPs are available but where regulatory or ethical oversight may be inferior to EU standards (15, 69). Overall, to maximize patient benefit, regulators should remain flexible and responsive, while developers must operate as efficiently as possible within the framework of current regulatory systems (35).

This environment can be overwhelming for academic researchers (15, 18, 51). In some cases, has even led ATMP developers to abandon their activities in the ATMP field (15, 50). To address these challenges, various solutions have been proposed. One suggestion is a revision of the ATMP Regulation with a stronger focus on delivering affordable therapies to all patients without necessarily requiring a full market entry pathway, thereby creating a more level playing field for public institutions and SMEs (15). Strengthening collaboration between academics, the CAT or the EMA, and national authorities, for example through sharing preclinical and clinical data to promote greater standardization and reduce costs has also been emphasized (32, 64). Another proposal involves harmonization among regulatory agencies, developing a minimum consensus package consisting of common scientific principles, general considerations, and core technical requirements that would apply broadly to most ATMPs, with add-on packages for individual cases (31, 53).

Additionally, the classification of ATMPs is not always straightforward. The mechanism of action is often unclear or multifactorial, and in some cases, it is difficult to determine whether a product should be considered an ATMP or a vaccine (38, 57). According to Pearce et al., the ATMP classification procedure in Europe is optional and non-binding, and there is no universal system for ATMP classification (38, 47, 48). Uncertainty in product classification can delay clinical trial planning and regulatory engagement, and in cases of misclassification may lead to significant loss of time and resources, potentially impacting or delaying the initiation of first-in-human studies.

It is also noteworthy that eight included articles reported that academic research teams lack regulatory expertise (18, 24, 38, 53, 59, 63, 66, 70). As potential solutions, these teams could build internal regulatory competences, seek advice from an external regulatory consultant, or engage in early dialog with regulatory authorities (24, 38, 53). Several studies reported that the centers that successfully achieved clinical translation were those that initiated early interactions with regulators (15, 48). However, these studies also noted that it remains unclear whether this advice is essential for trial initiation, or whether only centers that already possess sufficient regulatory competence are likely to engage with regulatory authorities in the first place (15, 48).

Importantly, the EU lags behind the United States in the number of ATMP clinical trials, both academic and industry-led (61). Comparisons between the EU and the US illustrate that the EU may be less competitive due to, for example, mandatory GMP requirements from phase I and the fact that clinical trial approval, monitoring, and evaluation occur at the level of national competent authorities (61). Those experts who remain in Europe tend to cluster in a limited number of strong life science hubs, further contributing to regional disparities in innovation capacity (61).

## 4 Discussion

ATMPs hold exceptional promise for addressing diseases with limited or no existing treatments, yet their successful translation to the clinic remains challenging. This scoping review mapped the regulatory hurdles that typically arise during the early stages of development or clinical translation and become apparent when transitioning to a clinical trial, as well as how these regulatory constraints shape other aspects of the development pathway. Together, these findings highlight persistent gaps in the ATMP development ecosystem, in part driven by the way regulatory requirements intersect with scientific, manufacturing and clinical challenges, thereby impeding timely and efficient clinical progress. In contrast to earlier narrative or phase-specific analyses, this review offers a structured synthesis of challenges encountered across the early ATMP development pathway, providing a more integrated perspective on the barriers faced by academic and SME developers. The challenges identified span four major domains: (A) preclinical evidence and product characterization, (B) GMP manufacturing, (C) clinical management and (D) regulatory requirements. These domains are highly interconnected, reinforcing each other, while regulatory requirements not only constitute a distinct domain but also influence and often amplify challenges arising across preclinical, manufacturing and clinical development.

Within the European context, the multitude and interrelated nature of these challenges indicate a misalignment between the requirements for ATMPs and the conventional regulatory framework. Importantly, while many of the identified challenges are intrinsic to ATMPs and are reported across different jurisdictions, their impact may be further compounded by the complexity and fragmentation of the EU regulatory landscape. According to one included article, there is currently limited evidence that certain regulatory standards consistently translate into improved product quality and patient safety,leading some authors to question how specific requirements, such as stringent GMP expectations, should be optimally applied in early development (15). The regulatory complexities and challenges associated with ATMP development are highlighted not only in the literature but also by the regulatory policy responses, through the establishment of ATMP-specific incentives (e.g., the academic ATMP pilot) and the EMA Action Plan for ATMPs, based on an EMA-hosted multi-stakeholder workshop, which aimed to develop multiple ATMP-specific guidelines, alongside numerous research infrastructures and consortia dedicated to ATMPs, including JOIN4ATMP, ESGCT and EuroGCT (1, 71). Together, these initiatives reflect not only support for innovation but also an institutional acknowledgement that conventional regulatory frameworks are insufficient to address the unique challenges posed by ATMPs.

Importantly, the EMA recognizes that academic ATMP developers often face difficulties with regulatory requirements, a situation made more complex by inconsistent regulations across different authorities, an issue our review clearly confirms (72). While early dialog with regulators is consistently highlighted as beneficial, a study from the STARS-consortium indicates that many groups do not initiate such interactions due to insufficient regulatory awareness or uncertainty regarding the expected level of preparedness (73, 74). Consequently, some promising academic ATMPs either do not reach clinical trial application or experience significant regulatory delays, making it difficult to secure and sustain funding during development. This challenge occurs because funding is often limited to academic or competitive public sources. Greater facilitation of private investment and spin-off models could help bridge this gap.

This study was comprised a scoping review, which enables broad and systematic mapping of the existing literature in a complex and evolving field such as ATMP development. This approach allows the inclusion of diverse types of evidence, including empirical studies, reviews, and perspective papers, thereby providing a comprehensive overview of reported challenges. However, several limitations should be acknowledged. First, as this methodology relies on published literature, it may not capture practical barriers that remain undocumented. Second, the available evidence base includes a substantial proportion of non-empirical publications, reflecting the limited number of empirical studies in this field. Therefore, empirical approaches, such as a survey or semi-structured interviews with ATMP developers, could complement these findings and provide additional insights. Finally, potential publication bias should be considered, as published experiences are more likely to originate from academic settings. However, this aligns with the focus of this study, as ATMP development, particularly in early stages, is predominantly driven by academic developers. Nevertheless, this may still result in an underrepresentation of perspectives from other stakeholders, such as SMEs or industry, and should be taken into account when interpreting the findings. Lastly, these findings could be further deepened by examining specific ATMP subtypes, as the nature and severity of hurdles may differ substantially between, for example, autologous and allogeneic products or between cell-based and vector-based therapies. Such stratified analyses would enable a more nuanced understanding of product-specific bottlenecks and support the development of tailored regulatory and translational guidance. Many included studies focused on non-genetically modified cell-based therapies classified as CTMPs; however, several identified manufacturing and regulatory challenges, such as variability in human starting materials complicating GMP compliance, are likely also relevant to GTMPs.

Recent regulatory developments by the EMA, such as the concept paper on the revision of GMP guidelines for ATMPs (EMA/INS/GMP/48771/2025) and the updated guideline on quality, non-clinical and clinical requirements for investigational ATMPs (EMA/CAT/22473/2025), aim to address several of the barriers identified in this review. For example, the GMP revision aims to reduce inconsistencies and better accommodate technological advances, by reinforcing a more systematic application of quality risk management principles and enabling the use of innovative manufacturing approaches. Similarly, the new ATMP guideline introduces an improved risk-based approach and provides more detailed guidance on data requirements for clinical trial applications, including risk-appropriate flexibilities. Importantly, the literature included in this review may predate the introduction of these updated regulatory frameworks, and therefore may not capture their potential impact on current development practices.

While these developments are important steps toward a more tailored regulatory framework for ATMPs, it remains uncertain to what extent they will fully resolve the challenges identified in this review, such as the operational complexity of GMP compliance, the variability in interpretation across Member States and limited regulatory expertise among academic developers. Future research such as qualitative studies (e.g., interviews with academic and SME developers) could provide insights into whether these new guidelines effectively alleviate existing bottlenecks or whether important gaps remain. This is particularly relevant in light of, IQVIA’s analysis indicating that Europe is seeing a decline in the number of clinical trials involving cell and gene therapies compared to other regions. To maintain its competitiveness, Europe must continue to assess how changing regulatory guidelines are addressing current challenges and identify any gaps where further support is needed.

## 5 Conclusion

ATMPs represent a rapidly advancing therapeutic field, yet multiple hurdles across preclinical development, manufacturing and regulatory navigation continue to slow their translation into clinical trials, especially for academic and SME developers with limited resources. This review underscores the need for clearer, harmonized and more flexible regulatory pathways in the EU, improved standards for quality and potency assessment, and enhanced regulatory support for academic developers. Initiatives such as JOIN4ATMP offer an opportunity to systematically address these barriers and help Europe develop a more coherent and supportive framework for ATMP clinical translation.
