# Supplementary material for: Why advanced therapy medicinal products struggle in clinical translation: an in-depth analysis of developmental challenges in the EU
Source: Front Med (Lausanne). 2026 Jun 2;13:1839531. doi: 10.3389/fmed.2026.1839531 (PMC13270379; doi:10.3389/fmed.2026.1839531)
Supplement: Supplementary file 1 [file Data_Sheet_1.PDF]

Supplementary Information I: PRISMA checklist for Scoping Reviews

Preferred Reporting Items for Systematic reviews and Meta-Analyses extension for Scoping Reviews (PRISMA-ScR) Checklist

SECTION	ITEM	PRISMA-ScR CHECKLIST ITEM	REPORTED ON PAGE #
**TITLE**			
Title	1	Identify the report as a scoping review.	1
**ABSTRACT**			
Structured summary	2	Provide a structured summary that includes (as applicable): background, objectives, eligibility criteria, sources of evidence, charting methods, results, and conclusions that relate to the review questions and objectives.	1
**INTRODUCTION**			
Rationale	3	Describe the rationale for the review in the context of what is already known. Explain why the review questions/objectives lend themselves to a scoping review approach.	2 - 3
Objectives	4	Provide an explicit statement of the questions and objectives being addressed with reference to their key elements (e.g., population or participants, concepts, and context) or other relevant key elements used to conceptualize the review questions and/or objectives.	3
**METHODS**			
Protocol and registration	5	Indicate whether a review protocol exists; state if and where it can be accessed (e.g., a Web address); and if available, provide registration information, including the registration number.	4
Eligibility criteria	6	Specify characteristics of the sources of evidence used as eligibility criteria (e.g., years considered, language, and publication status), and provide a rationale.	5
Information sources*	7	Describe all information sources in the search (e.g., databases with dates of coverage and contact with authors to identify additional sources), as well as the date the most recent search was executed.	4
Search	8	Present the full electronic search strategy for at least 1 database, including any limits used, such that it could be repeated.	Supplementary Information I
Selection of sources of evidence†	9	State the process for selecting sources of evidence (i.e., screening and eligibility) included in the scoping review.	4-5
Data charting process‡	10	Describe the methods of charting data from the included sources of evidence (e.g., calibrated forms or forms that have been tested by the team before their use, and whether data charting was done independently or in duplicate) and any processes for obtaining and confirming data from investigators.	6
Data items	11	List and define all variables for which data were sought and any assumptions and simplifications made.	6
Critical appraisal of individual	12	If done, provide a rationale for conducting a critical appraisal of included sources of evidence; describe the methods used and how this information was used in any data synthesis (if appropriate).	Click here to enter text.

sources of evidence§			
Synthesis of results	13	Describe the methods of handling and summarizing the data that were charted.	6
**RESULTS**			
Selection of sources of evidence	14	Give numbers of sources of evidence screened, assessed for eligibility, and included in the review, with reasons for exclusions at each stage, ideally using a flow diagram.	7
Characteristics of sources of evidence	15	For each source of evidence, present characteristics for which data were charted and provide the citations.	Supplementary Information III
Critical appraisal within sources of evidence	16	If done, present data on critical appraisal of included sources of evidence (see item 12).	Click here to enter text.
Results of individual sources of evidence	17	For each included source of evidence, present the relevant data that were charted that relate to the review questions and objectives.	8-18 + Supplementary Information
Synthesis of results	18	Summarize and/or present the charting results as they relate to the review questions and objectives.	8-18
**DISCUSSION**			
Summary of evidence	19	Summarize the main results (including an overview of concepts, themes, and types of evidence available), link to the review questions and objectives, and consider the relevance to key groups.	19
Limitations	20	Discuss the limitations of the scoping review process.	23
Conclusions	21	Provide a general interpretation of the results with respect to the review questions and objectives, as well as potential implications and/or next steps.	24
**FUNDING**			
Funding	22	Describe sources of funding for the included sources of evidence, as well as sources of funding for the scoping review. Describe the role of the funders of the scoping review.	Title page

JBI = Joanna Briggs Institute; PRISMA-ScR = Preferred Reporting Items for Systematic reviews and Meta-Analyses extension for Scoping Reviews.

* Where *sources of evidence* (see second footnote) are compiled from, such as bibliographic databases, social media platforms, and Web sites.

† A more inclusive/heterogeneous term used to account for the different types of evidence or data sources (e.g., quantitative and/or qualitative research, expert opinion, and policy documents) that may be eligible in a scoping review as opposed to only studies. This is not to be confused with *information sources* (see first footnote).

‡ The frameworks by Arksey and O'Malley (6) and Levac and colleagues (7) and the JBI guidance (4, 5) refer to the process of data extraction in a scoping review as data charting.

§ The process of systematically examining research evidence to assess its validity, results, and relevance before using it to inform a decision. This term is used for items 12 and 19 instead of "risk of bias" (which is more applicable to systematic reviews of interventions) to include and acknowledge the various sources of evidence that may be used in a scoping review (e.g., quantitative and/or qualitative research, expert opinion, and policy document).

From: Tricco AC, Lillie E, Zarin W, O'Brien KK, Colquhoun H, Levac D, et al. PRISMA Extension for Scoping Reviews (PRISMAScR): Checklist and Explanation. Ann Intern Med. 2018;169:467–473. doi: 10.7326/M18-0850.

Supplementary Information II: search string

<u>Embase (via Embase.com)</u>

Concept 1 = ATMPs

'advanced therapy'/exp OR 'advanced therapy medicinal product'/exp OR 'advanced therap*':ti,ab,kw OR ‘atmp$’:ti,ab,kw OR 'gene therapy'/de OR 'angiogenic gene therapy'/exp OR 'antiangiogenic gene therapy'/exp OR 'cancer gene therapy'/exp OR 'cardiovascular gene therapy'/exp OR 'cell based gene therapy'/exp OR 'gene replacement therapy'/exp OR 'genetic immunization'/exp OR 'germ line gene therapy'/exp OR 'nonviral gene therapy'/exp OR 'somatic gene therapy'/exp OR 'stem cell gene therapy'/exp OR 'viral gene therapy'/exp OR 'suicide gene therapy'/exp OR 'gene therapy medicinal product'/exp OR 'genetic immuni$ation':ti,ab,kw OR 'ribozyme therap*':ti,ab,kw OR 'gene therap*':ti,ab,kw OR 'genetic therap*':ti,ab,kw OR 'gene treatment*':ti,ab,kw OR ‘gtmp$’:ti,ab,kw OR ‘ctmp$’:ti,ab,kw OR 'regenerative medicine'/exp OR 'regenerative medicin*':ti,ab,kw OR 'regenerative therap*':ti,ab,kw OR 'somatic cell therapy'/exp OR 'somatic cell therap*':ti,ab,kw OR 'dna therap*':ti,ab,kw OR 'adoptive immunotherapy'/de OR 'adoptive immunotherap*':ti,ab,kw OR 'adoptive transfer'/exp OR ‘adoptive transfer$’:ti,ab,kw OR ‘adoptive cell$ transfer$’:ti,ab,kw OR 'til therapy'/exp OR 'til therap*':ti,ab,kw OR 'tumor infiltrating lymphocyte*':ti,ab,kw OR 'cellular immunotherapy'/exp OR 'cellular immunotherap*':ti,ab,kw OR 'chimeric antigen receptor immunotherapy'/exp OR 'chimeric antigen receptor':ti,ab,kw OR 'car cancer immunotherap*':ti,ab,kw OR 'car immunotherap*':ti,ab,kw OR 'car therap*':ti,ab,kw OR 'car t':ti,ab,kw OR 'ribozyme therapy'/exp OR 'ribozyme therap*':ti,ab,kw OR 'immunopotentiation':ti,ab,kw OR ‘cell transplantation’/exp OR ‘cell transplant*’:ti,ab,kw OR 'ex vivo expanded autologous human corneal epithelial cells containing stem cells':ti,ab,kw OR 'holoclar':ti,ab,kw OR 'imlygic':ti,ab,kw OR 'strimvelis':ti,ab,kw OR 'spherox':ti,ab,kw OR 'alofisel':ti,ab,kw OR 'yescarta':ti,ab,kw OR 'kymriah':ti,ab,kw OR 'luxturna':ti,ab,kw OR 'zolgensma':ti,ab,kw OR 'libmeldy':ti,ab,kw OR 'tecartus':ti,ab,kw OR 'abecma':ti,ab,kw OR 'breyanzi':ti,ab,kw OR 'carvykti':ti,ab,kw OR 'upstaza':ti,ab,kw OR 'roctavian':ti,ab,kw OR 'ebvallo':ti,ab,kw OR 'hemgenix':ti,ab,kw OR 'casgevy':ti,ab,kw OR 'durveqtix':ti,ab,kw OR 'talimogene laherparepvec':ti,ab,kw OR 'autologous cd34+ enriched cell fraction that contains cd34+ cells transduced with retroviral vector that encodes for the human ada cdna sequence':ti,ab,kw OR 'darvadstrocel':ti,ab,kw OR 'axicabtagene ciloleucel':ti,ab,kw OR 'tisagenlecleucel':ti,ab,kw OR 'voretigene neparvovec':ti,ab,kw OR 'onasemnogene abeparvovec':ti,ab,kw OR 'autologous cd34+ cells encoding arsa gene':ti,ab,kw OR 'brexucabtagene autoleucel':ti,ab,kw OR 'idecabtagene vicleucel':ti,ab,kw OR 'lisocabtagene maraleucel':ti,ab,kw OR 'ciltacabtagene autoleucel':ti,ab,kw OR 'eladocagene exuparvovec':ti,ab,kw OR 'valoctocogene roxaparvovec':ti,ab,kw OR 'tabelecleucel':ti,ab,kw OR 'etranacogene dezaparvovec':ti,ab,kw OR 'exagamglogene autotemcel':ti,ab,kw OR 'fidanacogene elaparvovec':ti,ab,kw OR ‘innovative treatment*’:ti,ab,kw OR ‘innovative medicine*’:ti,ab,kw OR ‘innovative drug*’:ti,ab,kw OR ‘innovative product*’:ti,ab,kw OR ‘innovative pharmaceutical*’:ti,ab,kw

Concept 2 = Developers

'drug industry'/exp OR ‘manufacturer$’:ti,ab,kw OR 'company':ti,ab,kw OR 'companies':ti,ab,kw OR 'industry':ti,ab,kw OR 'industries':ti,ab,kw OR ‘developer*’:ti,ab,kw OR ((‘micro’ NEXT/6 'enterprise'):ti,ab,kw) OR (('small' NEXT/6 'enterprise'):ti,ab,kw) OR (('medium' NEXT/6 'enterprise'):ti,ab,kw) OR ((‘micro’ NEXT/6 'enterprises'):ti,ab,kw) OR (('small' NEXT/6 'enterprises'):ti,ab,kw) OR (('medium' NEXT/6 'enterprises'):ti,ab,kw) OR 'academia':ti,ab,kw OR 'academic':ti,ab,kw OR ‘manufacturing’:ti,ab,kw OR ‘researcher$’:ti,ab,kw OR ‘innovator’/exp OR ‘innovator*’:ti,ab,kw

Concept 3 = regulatory

NOT ('conference abstract':it OR 'article in press':it OR 'short survey':it OR 'note':it OR 'letter':it OR 'editorial':it OR 'chapter':it OR 'erratum':it OR 'tombstone':it OR 'preprint':it)

Scopus (via Scopus.com)

Concept 1 = ATMPsc

TITLE-ABS(“advanced therap*” OR “ATMP*” OR “genetic immuni*ation” OR “ribozym therap*” OR “gene therap*” OR “genetic therap*” OR “gene treatment*” OR “GTMP*” OR “CTMP*” OR “regenerative medicin*” OR “regenerative therap*” OR “somatic cell therap*” OR “DNA therap*” OR “adoptive immunotherap*” OR “adoptive transfer*” OR “adoptive cell* transfer*” OR “TIL therap*” OR “tumor infiltrating lymphocyte*” OR “cellular immunotherap*” OR “chimeric antigen receptor” OR “CAR cancer immunotherap*” OR “CAR immunotherap*” OR “CAR therap*” OR “chimeric antigen receptor cancer immunotherap*” OR “chimeric antigen receptor engineered cancer immunotherap*” OR “CAR T” OR “mesenchymal stromal cell therap*” OR “ribozyme therap*” OR “immunopotentiation” OR “cell transplant*” OR “ex vivo expanded autologous human corneal epithelial cells containing stem cells” OR “Holoclar” OR “Imlygic” OR “Strimvelis” OR “Spherox” OR “Alofisel” OR “Yescarta” OR “Kymriah” OR “Luxturna” OR “Zolgensma” OR “Libmeldy” OR “Tecartus” OR “Abecma” OR “Breyanzi” OR “Carvykti” OR “Upstaza” OR “Roctavian” OR “Ebvallo” OR “Hemgenix” OR “Casgevy” OR “Durveqtix” OR “talimogene laherparepvec” OR “autologous CD34+ enriched cell fraction that contains CD34+ cells transduced with retroviral vector that encodes for the human ADA cDNA sequence” OR “spheroids of human autologous matrix-associated chondrocytes” OR “darvadstrocel” OR “axicabtagene ciloleucel” OR “tisagenlecleucel” OR “voretigene neparvovec” OR “onasemnogene abeparvovec” OR “Autologous CD34+ cells encoding ARSA gene” OR “Brexucabtagene autoleucel” OR “idecabtagene vicleucel” OR “lisocabtagene maraleucel” OR “ciltacabtagene autoleucel” OR “eladocagene exuparvovec” OR “Valoctocogene roxaparvovec” OR “tabelecleucel” OR “etranacogene dezaparvovec” OR “Exagamglogene autotemcel” OR “fidanacogene elaparvovec” OR “innovative treatment*” OR “innovative medicine*” OR “innovative drug*” OR “innovative product*” OR “innovative pharmaceutical*” OR “treatment paradigm*”) OR AUTHKEY(“advanced therap*” OR “ATMP*” OR “genetic immuni*ation” OR “ribozym therap*” OR “gene therap*” OR “genetic therap*” OR “gene treatment*” OR “GTMP*” OR “CTMP*” OR “regenerative medicin*” OR “regenerative therap*” OR “somatic cell therap*” OR “DNA therap*” OR “adoptive immunotherap*” OR “adoptive transfer*” OR “adoptive cell* transfer*” OR “TIL therap*” OR “tumor infiltrating lymphocyte*” OR “cellular immunotherap*” OR “chimeric antigen receptor” OR “CAR cancer immunotherap*” OR “CAR immunotherap*” OR “CAR therap*” OR “chimeric antigen receptor cancer immunotherap*” OR “chimeric antigen receptor engineered cancer immunotherap*” OR “CAR T” OR “mesenchymal stromal cell therap*” OR “ribozyme therap*” OR “immunopotentiation” OR “cell transplant*” OR “ex vivo expanded autologous human corneal epithelial cells containing stem cells” OR “Holoclar” OR “Imlygic” OR “Strimvelis” OR “Spherox” OR “Alofisel” OR “Yescarta” OR “Kymriah” OR “Luxturna” OR “Zolgensma” OR “Libmeldy” OR “Tecartus” OR “Abecma” OR “Breyanzi” OR “Carvykti” OR “Upstaza” OR “Roctavian” OR “Ebvallo” OR “Hemgenix” OR “Casgevy” OR “Durveqtix” OR “talimogene laherparepvec” OR “autologous CD34+ enriched cell fraction that contains CD34+ cells transduced with retroviral vector that encodes for the human ADA cDNA sequence” OR “spheroids of human autologous matrix-associated chondrocytes” OR “darvadstrocel” OR “axicabtagene ciloleucel” OR “tisagenlecleucel” OR “voretigene neparvovec” OR “onasemnogene abeparvovec” OR “Autologous CD34+ cells encoding ARSA gene” OR “Brexucabtagene autoleucel” OR “idecabtagene vicleucel” OR “lisocabtagene maraleucel” OR “ciltacabtagene autoleucel” OR “eladocagene exuparvovec” OR “Valoctocogene roxaparvovec” OR “tabelecleucel” OR “etranacogene dezaparvovec” OR “Exagamglogene autotemcel” OR “fidanacogene elaparvovec” OR “innovative treatment*” OR “innovative medicine*” OR “innovative drug*” OR “innovative product*” OR “innovative pharmaceutical*” OR “treatment paradigm*”)

Concept 2 = Developers

TITLE-ABS(“manufacturer*” OR “company” OR “companies” OR “industry” OR “industries” OR “developer*” OR (“micro” W/6 “enterprise”) OR (“small” W/6 “enterprise”) OR (“medium” W/6 “enterprise”)

OR (“micro” W/6 “enterprises”) OR (“small” W/6 “enterprises”) OR (“medium” W/6 “enterprises”) OR “academia” OR “academic” OR “manufacturing” OR “researcher*” OR “innovator*”) OR AUTHKEY(“manufacturer*” OR “company” OR “companies” OR “industry” OR “industries” OR “developer*” OR (“micro” W/6 “enterprise”) OR (“small” W/6 “enterprise”) OR (“medium” W/6 “enterprise”) OR (“micro” W/6 “enterprises”) OR (“small” W/6 “enterprises”) OR (“medium” W/6 “enterprises”) OR “academia” OR “academic” OR “manufacturing” OR “researcher*” OR “innovator*”)

Concept 3 = regulatory

TITLE-ABS(“legal” OR “legistlat*” OR “law” OR “laws” OR “regulat*” OR “clinical trial” OR “scientific advi*e” OR “SAWP” OR “prime scheme” OR (“european medicines” W/3 “agency”) OR “EMA” OR “food and drug administration” OR “FDA” OR “MHRA” OR “pharmaceuticals and medical devices agency” OR “PMDA” OR “early dialogue” OR “innovation task force” OR “ITF” OR “committee for medicinal products for human use” OR “CHMP” OR “committee for advanced therap*” OR “discussion meeting” OR (“translation” W/4 “gap”) OR (“translational” W/4 “gap”) OR (“translation” W/4 “gaps”) OR (“translational” W/4 “gaps”) OR “regulatory science*” OR “national competent authority”) OR AUTHKEY(“legal” OR “legistlat*” OR “law” OR “laws” OR “regulat*” OR “clinical trial” OR “clinical trial” OR “scientific advi*e” OR “SAWP” OR “prime scheme” OR (“european medicines” W/3 “agency”) OR “EMA” OR “food and drug administration” OR “FDA” OR “MHRA” OR “pharmaceuticals and medical devices agency” OR “PMDA” OR “early dialogue” OR “innovation task force” OR “ITF” OR “committee for medicinal products for human use” OR “CHMP” OR “committee for advanced therap*” OR “discussion meeting” OR (“translation” W/4 “gap”) OR (“translational” W/4 “gap”) OR (“translation” W/4 “gaps”) OR (“translational” W/4 “gaps”) OR “regulatory science*” OR “national competent authority”)

Pubmed (via Pubmed.com)

Concept 1 = ATMPs

“advanced therap*”[tiab] OR “atmp*”[tiab] OR “genetic therapy”[mesh] OR “genetic immunisation”[tiab] OR “genetic immunization”[tiab] OR “ribozyme therap*”[tiab] OR “gene therap*”[tiab] OR “genetic therap*”[tiab] OR “gene treatment*”[tiab] OR “gtmp*”[tiab] OR “ctmp*”[tiab] OR “regenerative medicine”[mesh] OR “regenerative medicin*”[tiab] OR “regenerative therap*”[tiab] OR “somatic cell therap*”[tiab] OR “dna therap*”[tiab] OR “adoptive immunotherap*”[tiab] OR "Immunotherapy, Adoptive"[Mesh] OR “adoptive transfer”[mesh] OR “adoptive transfer*”[tiab] OR “adoptive cell* transfer*”[tiab] OR “til therap*”[tiab] OR “tumor infiltrating lymphocyte*”[tiab] OR “cellular immunotherap*”[tiab] OR “chimeric antigen receptor”[tiab] OR “car immunotherap*”[tiab] OR “car therap*”[tiab] OR “car t”[tiab] OR “immunopotentiation”[tiab] OR “cell transplantation”[mesh] OR “cell transplant*”[tiab] OR “holoclar”[tiab] OR “imlygic”[tiab] OR “strimvelis”[tiab] OR “spherox”[tiab] OR “alofisel”[tiab] OR “yescarta”[tiab] OR “kymriah”[tiab] OR “luxturna”[tiab] OR “zolgensma”[tiab] OR “libmeldy”[tiab] OR “tecartus”[tiab] OR “abecma”[tiab] OR “breyanzi”[tiab] OR “carvykti”[tiab] OR “upstaza”[tiab] OR “roctavian”[tiab] OR “ebvallo”[tiab] OR “hemgenix”[tiab] OR “casgevy”[tiab] OR “talimogene laherparepvec”[tiab] OR “darvadstrocel”[tiab] OR “axicabtagene ciloleucel”[tiab] OR “tisagenlecleucel”[tiab] OR “voretigene neparvovec”[tiab] OR “onasemnogene abeparvovec”[tiab] OR “brexucabtagene autoleucel”[tiab] OR “idecabtagene vicleucel”[tiab] OR “lisocabtagene maraleucel”[tiab] OR “ciltacabtagene autoleucel”[tiab] OR “eladocagene exuparvovec”[tiab] OR “valoctocogene roxaparvovec”[tiab] OR “tabelecleucel”[tiab] OR “etranacogene dezaparvovec”[tiab] OR “exagamglogene autotemcel”[tiab] OR “fidanacogene elaparvovec”[tiab] OR “innovative treatment*”[tiab] OR “innovative medicine*”[tiab] OR “innovative drug*”[tiab] OR “innovative product*”[tiab] OR “innovative pharmaceutical*”[tiab]

Concept 2 = Developers

“drug industry”[mesh] OR “manufacturer*”[tiab] OR “company”[tiab] OR “companies”[tiab] OR “industry”[tiab] OR “industries”[tiab] OR “developer*”[tiab] OR “micro enterprise”[tiab:~5] OR “small enterprise”[tiab:~5] OR “medium enterprise”[tiab:~5] OR “micro enterprises”[tiab:~5] OR “small enterprises”[tiab:~5] OR “medium enterprises”[tiab:~5] OR “academia”[tiab] OR “academic”[tiab] OR “manufacturing”[tiab] OR “researcher*”[tiab] OR “innovator*”[tiab]

Concept 3 = regulatory

“legal”[tiab] OR “legistlat*”[tiab] OR “law”[tiab] OR “laws”[tiab] OR “regulat*”[tiab] OR “clinical trial”[tiab] OR “evidence gaps”[mesh]OR “legislation as topic”[mesh] OR “scientific advise”[tiab] OR “scientific advice”[tiab] OR “SAWP”[tiab] OR “prime scheme”[tiab] OR “european medicines agency”[tiab:~2] OR “EMA”[tiab] OR “United States Food and Drug Administration”[mesh] OR “food and drug administration”[tiab] OR “FDA”[tiab] OR “MHRA”[tiab] OR “pharmaceuticals and medical devices agency”[tiab] OR “PMDA”[tiab] OR “early dialogue”[tiab] OR “innovation task force”[tiab] OR “ITF”[tiab] OR “committee for medicinal products for human use”[tiab] OR “CHMP”[tiab] OR “committee for advanced therap*”[tiab] OR “discussion meeting”[tiab] OR “translation gap”[tiab:~3] OR “translational gap”[tiab:~3] OR “translation gaps”[tiab:~3] OR “translational gaps”[tiab:~3] OR "National Center for Advancing Translational Sciences (U.S.)"[Mesh] OR “national competent authority”[tiab]

Supplementary Information III: overview of included articles

	Title	Author	Publication year	Geographical region	Study design
**1**	A roadmap toward clinical translation of genetically-modified stem cells for treatment of HIV	Abou-El-Enein et al.	2014	EU and USA	Review
**2**	Regulation of advanced therapy medicinal products in Europe and the role of academia	Pearce et al.	2014	EU	Survey and interviews
**3**	Concise review: Making and using clinically compliant pluripotent stem cell lines	Carpenter et al.	2015	USA	Concise review
**4**	The translation of cell-based therapies: Clinical landscape and manufacturing challenges	Heathman and Nienow	2015	UK	Review
**5**	Adoptive cellular therapy: a race to the finish line	June et al.	2015	USA	Perspective
**6**	Ethics and policy issues for stem cell research and pulmonary medicine	Lowenthal and Sugarman	2015	USA	Review
**7**	Encountering Challenges with the EU Regulation on Advance Therapy Medical Products	Mansnérus	2015	EU	Review
**8**	Overcoming Challenges in Process Development of Cellular Therapies	Nordberg and Loboa	2015	USA	Perspective
**9**	Hurdles in therapy with regulatory T cells	Trzonkowski et al.	2015	EU	Perspective
**10**	Manufacture of tumor- and virus-specific T lymphocytes for adoptive cell therapies	Wang and Rivière	2015	USA	Review
**11**	Concise review: The U.S. food and drug administration and regenerative medicine	Witten et al.	2015	USA	Concise review
**12**	The early career researcher's toolkit: Translating tissue engineering, regenerative medicine and cell therapy products	Rafiq et al.	2015	EU	Perspective
**13**	Are there specific translational challenges in regenerative medicine? Lessons from other fields	Gardner et al.	2015	UK	Review
**14**	Recent policies that support clinical application of induced pluripotent stem cell-based regenerative therapies	Azuma and Yamanaka	2016	Japan	Review

**15**	Adapting Preclinical Benchmarks for First-in-Human Trials of Human Embryonic Stem Cell-Based Therapies	Barazzetti et al.	2016	EU and USA	Document analysis
**16**	Intraspinal stem cell transplantation for amyotrophic lateral sclerosis: Ready for efficacy clinical trials?	Atassi et al.	2016	EU, USA and UK	Workshop
**17**	Redirecting T cells with Chimeric Antigen Receptor (CAR) for the treatment of childhood acute lymphoblastic leukemia	Biondi et al.	2017	EU	Review
**18**	Regulatory and scientific advancements in gene therapy: State-of-the-art of clinical applications and of the supporting european regulatory framework	Carvalho et al.	2017	EU	Review
**19**	Innovative regenerative medicines in the EU: A better future in evidence?	Corbett et al.	2017	EU	Debate
**20**	Regulatory considerations for gene therapy products in the US, EU, and Japan	Halioua-Haubold et al.	2017	EU, USA and Japan	Review
**21**	Clinical development of CAR T cells—challenges and opportunities in translating innovative treatment concepts	Hartmann et al.	2017	EU	Review
**22**	New Japanese regulatory frameworks for clinical research and marketing authorization of gene therapy and cellular therapy products	Nagai and Ozawa	2017	Japan	Review
**23**	Stem cell therapy clinical research: A regulatory conundrum for academia	Nagpal et al.	2017	EU, USA, Japan, Canada, Australia	Review
**24**	Scientific considerations for the regulatory evaluation of cell therapy products	Petricciani et al.	2017	Global	Review
**25**	Gene delivery to the lungs: pulmonary gene therapy for cystic fibrosis	Villate-Beitia et al.	2017	EU	Review
**26**	A Guide to Approaching Regulatory Considerations for Lentiviral-Mediated Gene Therapies	White et al.	2017	EU and UK	Review
**27**	"Can harmonized regulation overcome intra-European differences? Insights from a European Phase III stem cell trial "	Hauskeller	2017	UK	Survey and interviews
**28**	Concise review: Mesenchymal stem cell therapy for pediatric disease: Perspectives on success and potential improvements	Nitkin and Bonfield	2017	USA	Concise review

**29**	Clinical Development and Commercialization of Advanced Therapy Medicinal Products in the European Union: How Are the Product Pipeline and Regulatory Framework Evolving?	Boráň et al.	2017	EU	Review
**30**	Clinical translation and regulatory aspects of CAR/TCR-based adoptive cell therapies—the German Cancer Consortium approach	Krackhardt et al.	2018	EU	Review
**31**	Cell and gene therapies: European view on challenges in translation and how to address them	Rousseau et al.	2018	EU	Review
**32**	Challenges in Advanced Therapy Medicinal Product Development: A Survey among Companies in Europe	ten Ham et al.	2018	EU	Survey
**33**	Identifying and Managing Sources of Variability in Cell Therapy Manufacturing and Clinical Trials	Silverman et al.	2019	USA	Review
**34**	Innovation in Chemistry, Manufacturing, and Controls—A Regulatory Perspective From Industry	Cauchon et al.	2019	USA	Review
**35**	A blueprint for translational regenerative medicine	Armstrong et al.	2020	UK	Review
**36**	Cell therapies for spinal cord injury: Trends and challenges of current clinical trials	Bartlett et al.	2020	Global	Review
**37**	Preclinical development of autologous hematopoietic stem cell-based gene therapy for immune deficiencies: A journey from mouse cage to bed side	Garcia-Perez et al.	2020	EU	Review
**38**	The Confluence of Innovation in Therapeutics and Regulation: Recent CMC Considerations	Gutierrez et al.	2020	USA	Review
**39**	Advanced Therapy Medicinal Products Challenges and Perspectives in Regenerative Medicine	Goula et al.	2020	EU	Review
**40**	Regulatory Framework for Academic Investigator-Sponsored Investigational New Drug Development of Cell and Gene Therapies in the USA	Dasgupta et al.	2021	USA	Review
**41**	Transitioning From Preclinical Evidence to Advanced Therapy Medicinal Product: A Spanish Experience	Gastelurrutia et al.	2021	EU	Review
**42**	Regulatory considerations for developing a phase I investigational new drug application for autologous induced pluripotent stem cells-based therapy product	Jha et al.	2021	USA	Review
**43**	CRISPR-derived genome editing therapies: Progress from bench to bedside	Rees et al.	2021	USA	Review

**44**	Towards a better use of scientific advice for developers of advanced therapies	Tavridou et al.	2021	EU	Review
**45**	Cell therapies in the clinic	Wang et al.	2021	Global	Review
**46**	Nonclinical safety assessment of engineered T cell therapies	Lebrec et al.	2021	USA	Workshop
**47**	Chimeric Antigen Receptor Immunotherapy for Solid Tumors: Choosing the Right Ingredients for the Perfect Recipe	Castiello et al.	2022	EU	Review
**48**	A Regulatory Risk-Based Approach to ATMP/CGT Development: Integrating Scientific Challenges With Current Regulatory Expectations	Salazar-Fontana	2022	EU and USA	Perspective
**49**	ATMP development and pre-GMP environment in academia: a safety net for early cell and gene therapy development and manufacturing	Silva et al.	2022	EU	Review
**50**	Recommendations for procurement of starting materials by apheresis for advanced therapy medicinal products	Manson et al.	2022	UK	Survey
**51**	Insights into the clinical development of regenerative medical products through a comparison of three cell-based products recently approved for limbal stem cell deficiency	Aketa et al.	2023	Japan	Review
**52**	Barriers to Treg therapy in Europe: From production to regulation	Hennessy et al.	2023	UK	Review
**53**	Key challenges in developing a gene therapy for Usher syndrome: machine-assisted scoping review	Bhat et al.	2024	EU	Review
**54**	Clinical translation of pluripotent stem cell-based therapies: successes and challenges	Christiansen and Kirkeby	2024	EU	Review
**55**	Scaling of cell and gene therapies to population	Knöbel and Bosio	2024	EU	Review
**56**	Academic challenges on advanced therapy medicinal products' development: a regulatory perspective	Olesti et al.	2024	EU	Review
**57**	Pharmaceutical and clinical challenges of biological medicines: ongoing hurdles from drug development to therapeutic applications	Paul and Sarkar	2024	USA	Review

**58**	Improving the Assessment of Risk Factors Relevant to Potential Carcinogenicity of Gene Therapies: A Consensus Article	Klapwijk et al.	2024	EU, USA, Japan and Australia	FGD
